# Cocktail sedation containing propofol versus conventional sedation for ERCP: a prospective, randomized controlled study

**DOI:** 10.1186/1471-2253-12-20

**Published:** 2012-08-09

**Authors:** Phonthep Angsuwatcharakon, Rungsun Rerknimitr, Wiriyaporn Ridtitid, Pradermchai Kongkam, Sahadol Poonyathawon, Yuwadee Ponauthai, Sakolkan Sumdin, Pinit Kullavanijaya

**Affiliations:** 1 Division of Gastroenterology, Department of Medicine, Faculty of Medicine, Chulalongkorn University, Bangkok, 10310, Thailand; 2 Department of Anesthesiology, Faculty of Medicine, Chulalongkorn University, Bangkok, 10330, Thailand

**Keywords:** Cocktail sedation containing propofol, Meperidine, Midazolam, ERCP

## Abstract

**Background:**

ERCP practically requires moderate to deep sedation controlled by a combination of benzodiazepine and opiod. Propofol as a sole agent may cause oversedation. A combination (cocktail) of infused propofol, meperidine, and midazolam can reduce the dosage of propofol and we hypothesized that it might decrease the risk of oversedation. We prospectively compare the efficacy, recovery time, patient satisfactory, and side effects between cocktail and conventional sedations in patients undergoing ERCP.

**Methods:**

ERCP patients were randomized into 2 groups; the cocktail group (n = 103) and the controls (n = 102). For induction, a combination of 25 mg of meperidine and 2.5 mg of midazolam were administered in both groups. In the cocktail group, a bolus dose of propofol 1 mg/kg was administered and continuously infused. In the controls, 25 mg of meperidine or 2.5 mg/kg of midazolam were titrated to maintain the level of sedation.

**Results:**

In the cocktail group, the average administration rate of propofol was 6.2 mg/kg/hr. In the control group; average weight base dosage of meperidine and midazolam were 1.03 mg/kg and 0.12 mg/kg, respectively. Recovery times and patients’ satisfaction scores in the cocktail and control groups were 9.67 minutes and 12.89 minutes (*P* = 0.045), 93.1and 87.6 (*P* <0.001), respectively. Desaturation rates in the cocktail and conventional groups were 58.3% and 31.4% (*P* <0.001), respectively. All desaturations were corrected with temporary oxygen supplementation without the need for scope removal.

**Conclusions:**

Cocktail sedation containing propofol provides faster recovery time and better patients’ satisfaction for patients undergoing ERCP. However, mild degree of desaturation may still develop.

**Trial registration:**

ClinicalTrials.gov, NCT01540084

## Background

In order to facilitate a high success rate and avoids patients’ discomfort from a long procedure, endoscopic retrograde cholangiopancreatography (ERCP) requires a good moderate to deep level of sedation [1,2]. Propofol has been increasingly used for sedation in many gastrointestinal procedures including ERCP because it has a shorter both half-life and results in a shorter recovery time than conventional sedation (opioid and/or benzodiazepine)[3-6]. In addition, it can be safely administered by non-anesthetist e.g. well-trained nurses with or without endoscopist’s advice [7-9]. Because of its narrow therapeutic window, the level of conscious sedation can easily go deeper from moderately deep sedation to near general anesthesia. Therefore, propofol as a sole agent can cause oversedation and apnea [4,10,11]. A combination of benzodiazepine, and propofol has been shown to reduce the dose requirement of propofol while maintaining comparable efficacy [12,13]. This in turn may reduce the risk of oversedation by propofol [14,15]. Moreover, at a moderate sedation level, propofol does not have analgesic property, patients undergoing ERCP under propofol sedation alone could be suffered from pain. A cocktail regimen containing meperidine/midazolam and propofol may add additive analgesic effect during ERCP. Furthermore, administering propofol by infusion pump provides constant plasma level of propofol and may reduce adverse effect of overdosing by a bolus dose. However, data for cocktail sedation in ERCP is limited to only a bolus propofol dosing [16,17].

This study was aimed to compare the differences in terms of recovery time, patients’ satisfaction, and sedation-related adverse events between conventional sedation (meperidine and midazolam), and cocktail regimen (meperidine, midazolam and continuously infused propofol) in patients undergoing ERCP.

## Methods

### Patients

Patients with suspected common bile duct stone(s) or distal common bile duct obstruction scheduled for ERCP during December 2006 to August 2009 at King Chulalongkorn Memorial hospital were recruited. Patients who were under 18 years old, pregnant; with American Society of Anesthesiologists’ (ASA) physical classification IV – V, with history of sulfite, egg or soy bean allergy, with emergency need for ERCP, those who informed consent could not be obtained, and those with possible complex ERCP (hilar cholangiocarcinoma, post Billroth II anatomy, etc. ) were excluded from this study. Verbal and written informed consent was obtained from all subjects. The randomization was done with a random sequence generated by a computer. Concealed envelop was broken when the patient was in the ERCP room. ERCP procedures were performed by an experienced endoscopist who performed 300–400 ERCPs annually for 10 years. The patients were put in a prone position during ERCP. The study protocol was approved by the Chulalongkorn Medical Institutional Review Board.

### Sedation and monitorings

During the procedure, patients’ conscious level, unexpected movement, blood pressure, heart rate and oxygen saturation were monitored by an experienced endoscopy nurse under supervision of an advanced endoscopy fellow who certified in advanced cardiopulmonary life support. Blood pressure was measured by an automated blood pressure cuff every 5 minutes. Oxygen saturation and heart rate were monitored by a pulse oxymetry machine. Respiratory effort and respiratory rate were observed by visual inspection. All records were done at baseline, just before starting sedation, and every five minutes thereafter until patient had a full recovery. When desaturation or apnea developed between the 5-minute interval, those parameters were additionally noted. Sedation was maintained at the level of moderate (purposeful response to verbal or tactile stimulation) to deep sedation (purposeful response to repeated or painful stimulation)[1]. The oxygen supplementation was given only when patients’ oxygen saturation dropped to less than 90% and lasted for more than 10 seconds. Resuscitation equipments, flumazenil and naloxone were available in the endoscopy unit.

After the procedure, patients were monitored for recovery scores (modified Aldrete score) [18]. Recovery scores were calculated at every 15 minutes. Time to full recovery was noted as the interval of 15 minutes. During the first 15 min, a nurse monitored patient closely at bedside and the exact time of full recovery in patients with fast recovery was noted.

### Outcome measurements and definitions

The nurses in the recovery room were blinded for type of sedation. The procedure-related time included (1) induction time (the time from sedation to scope intubation), (2) procedural time (the time from scope intubation to scope withdrawal), and (3) recovery time (the time from scope withdrawal to full recovery (modified Aldrete score of 10)). The induction time and procedural time were recorded by the nurse in the endoscopy unit. The recovery time was recorded by the nurse in the recovery room.

Cardiopulmonary complications included (1) hypotension (systolic blood pressure drops to less than 90 mmHg or decreases more than 25% from the baseline), (2) bradycardia (heart rate <50 beats/min), (3) desaturation (oxygen saturation <90% for >10 seconds), and (4) apnea (cessation of respiratory activity for over 10 seconds under visual observation). If the oxygen saturation dropped to <85% for more than 30 seconds despite oxygen supplementation or apnea occurred, the procedure would be interrupted and reversal medications would be given to the patient.

### Medications

Endoscopic nurse who well trained in propofol administration, administered medications and continuously monitored patients in both groups under supervision of an advanced endoscopy fellow who certified in advanced cardiac life support. All dosages were adopted from our previous publication [6] and our previous experience in using these agents. For induction, 25 mg of meperidine and 2.5 mg of midazolam were administered in both groups. In the cocktail group, one milligram per kilogram body weight of 1% propofol emulsion (Baxter Healthcare Corp., Irvine, CA) was slowly infused by an automated pump (Terufusion syringe pump TE-331, Terumo Corporation, Tokyo, Japan). To maintain conscious level of patient in the conventional group to be at moderate or deep level, 25 mg of meperidine and/or 2.5 mg of midazolam were administered as necessary, whereas patients in the cocktail group were continuously administered with 1% propofol at the rate of 1 mg/kg/hr. An additional 0.5 mg/kg bolus was administered as needed to achieve the designed conscious level.

### Patient assessments

After patients gained a full level of consciousness, the independent nurse in recovery room who blinded to the randomization assessed patients’ discomfort, gagging, and satisfaction by using a visual analogue scale (VAS). The VAS range from 0–100 for discomfort (0: none;100 worst discomfort imaginable, gagging (0: none; 100: worst gagging imaginable), and satisfaction (0: not satisfied at all; 100: completely satisfied).

### Statistical analysis

Sample sizes were calculated based on mean and standard deviation of recovery times in the convention groups according to the results from our previous study; 34.25 minutes and 16.06, respectively [6]. To determine the difference by two sided test with type I error of 0.05, power of 85%, 20% reduction of recovery time, and the ratio of study to control groups of 1, 100 subjects were needed from each groups.

Continuous outcomes with normal distribution were analyzed with independent 2 sample *t*-test, and Wilcoxon sum rank test was used to analyze nonparametric data. Categorical outcomes were analyzed with Chi-square or Fisher’s exact test where appropriate. Reported *P* value was 2-sided, with value of ≤ 0.05 considered statistically significant. The SPSS software version 16.0 (SPSS Inc., Chicago, IL, USA) was used to analyzed the outcome.

## Results

### Patient and procedural variables

Two hundred and five patients were enrolled in the study. One hundred and two patients and 103 patients were randomly assigned into the conventional group and the cocktail group respectively. The baseline characteristics of patients including gender, age, type of ERCP, body mass index (BMI) and ASA physical status classification were not different (Table 1). The dosage of meperidine and midazolam in the conventional group were 2.42 and 0.29 mg/kg/hr, respectively, whereas the dosage of meperidine, midazolam and propofol in the cocktail group were 1.06, 0.11, and 6.18 mg/kg/hr, respectively. Induction times in the convention group and the cocktail group were 3.10 and 3.68 minutes, respectively. Procedural times in the convention group and the cocktail group were 31.60 and 27.88 minutes, respectively. These results were not different between the two groups (Table 2). Indications for ERCP and difficulty grade of ERCP were not different between the two groups (Table 2).

**Table 1:** Patient Characteristics

	**Conventional group**	**Cocktail group**	**P**
Gender (male/female)	51/51	52/51	1.000
Age (years) ^a^	57.27 ± 14.52	59.56 ± 13.65	0.246
Type of ERCP			0.877
- No-therapy*	17 (16.7%)	20 (18.4%)	
- Therapy	83 (81.4%)	81 (78.6%)	
- Fail	2 (1.9%)	2 (1.9%)	
BMI ^a^	22.18 ± 3.62	23.06 ± 3.83	0.097
ASA class			0.630
- I	42 (41.2%)	49 (47.6%)	
- II	49 (48%)	43 (41.7%)	
- III	11 (10.8%)	11 (10.7%)	

^a^ Data given as mean ± SD.

*No-therapy = cholangiogram and pancreatogram were obtained by contrast injection and no treatment was required after the negative result of cholangio-pancreatogram.

**Table 2:** Procedural Data

	**Conventional group**	**Cocktail group**	**P**
Mean dosage (mg) ^a^			N/A
- Meperidine	58.33 ± 24.46	25	
- Midazolam	6.81 ± 2.12	2.5	
- Propofol	0	172.08 ± 92.15	
Dosage (mg/kg/hr) ^a^			N/A
- Meperidine	2.42 ± 2.58	1.06 ± 0.60	
- Midazolam	0.29 ± 0.36	0.11 ± 0.06	
- Propofol	0	6.18 ± 3.0	
Indications for ERCP			0.621
- CBD stone	51 (50.0%)	53 (51.5%)	
- Benign biliary stricture	7 (6.9%)	10 (9.7%)	
- Malignant biliary stricture	28 (27.4%)	20 (19.4%)	
- Pancreatic therapy	5 (4.9%)	8 (7.8%)	
- Others	11 (10.8%)	12 (11.6)	
Time to intubation (min) ^a^	3.10 ± 1.86	3.68 ± 2.38	0.053
Duration (min) ^a^	31.60 ± 17.56	27.88 ±14.38	0.099

^a^ Data given as mean ± SD.

### Cardiovascular and respiratory parameters

Cardiovascular and respiratory data are shown in Table 3. Baseline systolic blood pressure (SBP) in the convention group and the cocktail group were 137.2 ± 22.1 and 134.2 ± 19.1 mmHg, respectively (*p* = 0.309). Baseline heart rate (HR) in the convention group and the cocktail group were 86.0 ± 14.8 and 85.7 ± 15.1 beats/minute, respectively (*p* = 0.867). Baseline oxygen saturation (SpO_2_) in the convention group and the cocktail group were 98.5 ± 1.5 and 97.8 ± 2.9%, respectively (*p* = 0.052). The mean percentage of dropping in SBP in the convention group and the cocktail group were 16.0 ± 10.5 and 17.2 ± 11.9%, respectively (*p* = 0.442). Percentage of patients with SBP dropping more than 25% from the baseline in the convention group and the cocktail group were 22.5 and 26.5%, respectively (*p* = 0.541). Five patients (4.9%) in the convention group and 14 patients (13.6%) in the cocktail group experienced hypotension, (*p* = 0.057). One patient in the cocktail group experienced bradycardia, which was transient and rescued by temporary cessation of propofol infusion, but the procedure was successfully continued without interruption. Heart rate decrease more than 25% from baseline developed in 17.6% and 23.3% of conventional and cocktail group, respectively (*p* = 0.316). Oxygen saturation of less than 90% and 85% were found more in the cocktail group than in the conventional group (58% vs. 31%; *p* < 0.001 and 9% vs. 1%; *p* = 0.019, respectively). However, desturation episodes were transient and all were corrected by patient arousal with temporary oxygen supplement. No reversal medication was required and the procedure was interrupted temporarily but no need for a scope withdrawal. After the patient regained normal oxygen saturation level without the need for oxygen supplement, the procedure was continued until finish. There was no apnea occurred in any patients.

**Table 3:** Cardiovascular and respiratory parameters

	**Conventional group**	**Cocktail group**	**P**
Baseline SBP (mmHg)^a^	137.2 ± 22.1	134.2 ± 19.1	0.309
Mean% decrease of SBP^a^ from baseline	16.0 ± 10.5	17.2 ± 11.9	0.442
SBP decrease ≥25% from baseline (% of patients)	22.5	26.2	0.541
SBP <90 mmHg (% of patients)	4.9	13.6	0.057
Baseline heart rate (HR/min) ^a^	86.0 ± 14.8	85.7 ± 15.1	0.867
Mean% decrease of HR from baseline ^a^	12.5 ± 11.3	14.1 ± 12.3	0.333
HR decrease ≥25% from baseline (% of patients)	17.6	23.3	0.316
Bradycardia (% of patients)	0	1	1.000^b^
Apnea	0	0	
Baseline SpO_2_ (%)^a^	98.5 ± 1.5	97.8 ± 2.9	0.052
Greatest decline of SpO_2_ from baseline ^a^	6.7 ± 6.1	8.7 ± 6.9	0.026
SpO2 <90% (% of patients, any episodes)	31.4	58.3	<0.001
SpO2 <85% (% of patients, any episodes)	1.0	8.7	0.019^b^

^a^ Data given as mean ± SD, ^b^ Fisher’s exact test.

### Recovery, patient tolerance and satisfaction

Recovery time, patients’ tolerance and satisfaction data are shown in Tables 4 and 5. Mean recovery times in the cocktail group and the conventional group were different significantly (9.7 min vs. 12.9 min; *p* = 0.045). Conscious score at 15, 30, 45 and 60 minutes were not different between the two groups. There were significantly differences of discomfort and gagging between both groups (*p* = 0.006 for discomfort and *p* = 0.003 for gagging). Satisfaction scores in the cocktail group and the conventional group were 93.1 and 87.6 respectively, the difference was also statistically significant (p < 0.001).

**Table 4:** Recovery parameters

	**Conventional group**	**Cocktail group**	**P**
Recovery time (min)	12.89 ± 12.87	9.67 ± 4.86	0.045
Conscious score at 15 min	9.73 ± 0.45	9.85 ± 0.53	0.102
Conscious score at 30 min	9.95 ± 0.21	9.99 ± 0.10	0.157
Conscious score at 45 min	9.99 ± 0.11	10.00 ± 0.00	0.320
Conscious score at 60 min	9.99 ± 0.11	10.00 ± 0.00	0.320

All data given as mean ± SD.

**Table 5:** Patient tolerance and satisfaction

	**Conventional group**	**Cocktail group**	**P**
Discomfort (Median; inter-quartile)	10 (10, 20)	10 (0, 20)	0.006^a^
Gagging (Median; inter-quartile)	10 (10, 20)	10 (0, 20)	0.003^a^
Satisfaction	87.6 ± 9.6	93.1 ± 9.1	<0.001

^a^ Mann–Whitney *U* test.

## Discussion

Over the last decade, we have seen an increase in propofol use for sedation in many GI endoscopies [2,3]. The most important advantage of this agent is its fast recovery profile. In addition, non-anesthetists are able to give this agent. However, its narrow therapeutic window with a possible risk of apnea is the main concern. Thus, adequate experience of those who can administer this agent and early detection of overdosing skill are mandatory for patients’ safety. Propofol sedation at a moderate level is safe and well accepted for many diagnostic GI procedures [1-3]. However, the risk will increase if the level of conscious sedation is designed at moderate to deep sedation. Practically, this level of sedation is preferred for ERCP sedation. Our study confirmed the benefit of cocktail sedation containing propofol infusion over the conventional sedation in many categories including recovery profiles, patients’ tolerance, and satisfaction. Another hypothetical benefit in the present study was decreasing the rate of desaturation by reducing propofol dosage. We hypothesized that by giving continuous perfusion of propofol, the constant level of propofol would not exceed the therapeutic window and this in turn might result in an acceptable desaturation rate that comparable to the conventional sedation. Although, the dosage of proposal used in this study was lower than that of propofol infusion alone in the same population reported by us previously (6.2 mg/kg/hr vs. 9.4 mg.kg.hr) [6]. Unfortunately, the present study failed to demonstrate the lower incidence of desaturation of this regimen over the conventional sedation. We observed a higher percentage of desaturation in the cocktail group than the conventional group (SpO2 <90%; 58.3% vs. 31.4%, *p* <0.01 and SpO2 < 85%; 8.7% vs. 1.0%, *p* < 0.019). However, these desaturation episodes were very short and did not interfere the continuity of our ERCP procedures. It may be implicated that reducing the dose of propofol was not equivalent to decreasing the risk of oversedation. We speculate that there are some pitfalls in our study that require further investigation. First, prophylactic oxygen supplementation was not routinely given to any of our patients. During the study period, all desaturated patients recovered immediately after oxygen cannula was given and only 2 L/min was required. There was no need for assisted ventilation in any patients. Other previous studies that gave a routine oxygen supplementation showed a lower rate of desaturation in the propofol group (5-24%) [12,13,17]. A prophylactic oxygen supplementation may apply to prevent this adverse event [19], however its discriminate use may expose patients to delays in the diagnosis of disventilation by maintaining apneic oxygenation during hypoventilation [20]. Second, meperidine as the choice of narcotic agent in this study may result in a higher rate of desaturation than other short acting and milder narcotic agents such as pentazocine and fentanyl. Pentazocine is a synthetic narcotic that designed to have a lower rate of respiratory suppression [21]. Ong, et al. used a combination of pentazocine and propofol for sedation in ERCP patients [17]. They demonstrated a lower rate of desaturation (<5%) than ours. In addition, fentanyl is another narcotic with shorter half-life than meperidine. When compared with conventional sedation, a combination of propofol and fentanyl resulted in a faster recovery with similar safety even when these combinations were given in cirrhotic patients who underwent upper GI endoscopy [22]. Lastly, hypercapnia usually develops before desaturation occurs [23]. If we have used capnography in our study, we might have detected earlier cases of propofol overdose and desaturation rate might have been lower. Qadeer, et al. demonstrated that capnography is helpful for a better detection of apnic episode when compared with visual respiratory observation as confirmed by a lower hypoxic rate in the patients who underwent ERCP/EUS with capnography on board [24].

Cardiovascular adverse event related to propofol based sedation is another concern in non-anesthetist administered technique. Although, we observed a higher rate of dropping in systolic blood pressure to less than 90 mmHg in the cocktail group than the conventional group (14% vs. 5%), this rate of hypotension was not statistically significant and still comparable to the previous studies (13%) [5,6]. Fortunately, these hypotensive episodes did not lead to any significant clinical cardiovascular compromise. We speculate that, histamine release causing hypotension can occur after a rapid administration of meperidine [21]. Propofol itself can also cause hypotesion by relaxing vascular smooth muscle [25]. Thus, the combined hypotensive effect may be potent especially during the initial phase of sedation. Alternatively, cocktail sedation containing fentanyl may cause a lower rate of hypotensive effect since fentanyl does not cause an over-release of histamine. However, further comparison study is required to confirm this hypothesis.

One of the limitations is that the level of sedation on each patient was controlled by a sedation nurse who was unblinded to sedative agents. Perhaps, there might be a bias in giving sedative agents at the doses that she preferred to be safe. However, this was more practical to real life practice since the level of sedation must be adjusted to the interest of patient safety as the first priority.

Although, we observed that the recovery time in the present conventional group (12.89 ± 12.87 minutes) seemed to be shorter than previous studies including our previous study (29–70 minutes) [4-6,10,25], the present study showed that the recovery time in the cocktail group was still significantly faster than the conventional group (9.67 ± 4.86 minutes vs. 12.89 ± 12.87 minutes, *p* = 0.045).We speculate that the overall shorter in procedural time was because of only non-complex ERCP such as common bile duct stone and distal common bile duct obstruction were recruited in the study. The low level of complexity in this seires was confirmed by a very low number of failed ERCP in both groups (0-2%).

Our patients in the cocktail group reported a significant higher satisfaction score than the patients in the conventional group (93 vs. 88, *p* < 0.001). The result was in line with a study by Lee, et al. that showed a non-significantly better satisfaction by patients when cocktail regimen was used for ERCP [16]. Moreover, their study demonstrated a significantly better satisfaction by both endoscopists /nurses when propofol was used for sedation. During our study, we did not encounter any undesirable movement that would interfere the procedure outcome or delay the procedure time of any of the patients from both groups. However, the limitation in our study is that our protocol did not have a formal intra-procedural assessment by sedation team. Therefore, a complete evaluation of patients’ cooperation may not be assessed from this study.

## Conclusion

In conclusion, the present study shows the superiority of cocktail sedation containing propofol infusion, midazolam, and meperidine over conventional sedation in terms of recovery profiles, patients’ tolerance, and satisfaction. Although there was no serious adverse event in the cocktail group, the results of the study actually showed the cocktail sedation increased the rate of desaturation, which implicated that reducing the dose of propofol in this cocktail protocol was not equivalent to decreasing the risk of oversedation. Adjustment in the protocol that may overcome this problem is required. We propose on using fentanyl or pentazocine instead of meperidine in the regime. Lastly, a prophylactic oxygen supplementation and monitoring with capnography may be of help.

## Competing interests

All authors have no financial and non-financial competing interests.

## Authors’ contributions

PA participated in the design of the study, patient recruitment, statistical analysis, and manuscript preparation. RR participated in patient recruitment, performing ERCP, and manuscript preparation. WR participated in patient recruitment, randomization and manuscript preparation. PKo participated in the design of the study, patient recruitment and manuscript review. SP participated in the design of the study and manuscript review. YP participated in sample processing, administration of study medications and manuscript review. SS participated in sample processing, monitor of patients after ERCP and manuscript review PK participated in study design and manuscript review. All authors read and approved the final manuscript.

## Pre-publication history

The pre-publication history for this paper can be accessed here:

http://www.biomedcentral.com/1471-2253/12/20/prepub
